# Research on the balanced, coordinated and sustainable development of China manufacturing industry

**DOI:** 10.1371/journal.pone.0322400

**Published:** 2025-04-28

**Authors:** Weiwei Zhu, Guozhuo Yang

**Affiliations:** School of Statistics and Data Science, Lanzhou University of Finance and Economics, Lanzhou, China; Southwestern University of Finance and Economics, CHINA

## Abstract

China’s manufacturing industry faces the multiple goals of balanced, coordinated and sustainable development. This paper clarifies the connotation of balanced, coordinated and sustainable development of the manufacturing industry from regional structure, industrial structure and development structure. The level of balanced, coordinated and sustainable development of the manufacturing industry is measured using various methods such as index construction model, coupled coordination model and objective assignment method. The temporal and spatial evolution characteristics of the balanced, coordinated and sustainable development of the manufacturing industry are analysed. The following conclusions were obtained: the overall level of manufacturing equilibrium in the east is high, and the level of manufacturing equilibrium in the west and northeast is low. Therefore, it is necessary to promote the level of manufacturing development in the central and western regions through industrial transfer and other means. The overall coordination level of manufacturing industry shows a clear upward trend. The coordination level of manufacturing industry in the east ranks first among the four regions, and the coordination level of manufacturing industry in the west has made the most obvious progress. The overall level of sustainable development of the manufacturing industry is on an upward trend, with the highest level of sustainable development of the manufacturing industry in the east and a relatively low level of sustainable development in the west. There is a need to achieve sustainable development of the manufacturing industry by promoting the integration and development of the digital economy and the manufacturing industry.

## 1. Introduction

China’s manufacturing sector has a long-standing imbalance between regional and industrial structures. The eastern coastal areas are concentrated in high-end manufacturing and export-oriented industries, while the central and western regions are still mainly resource-intensive [1,2]. This unbalanced pattern has led to limited technological spillovers, duplicated construction and increased overcapacity. Research on the balanced development of manufacturing industry can help optimise the allocation of production factors and avoid the ‘siphon effect’ that weakens the competitiveness of less developed regions [3,4]. It plays a fundamental role in the construction of a unified domestic market and the promotion of the ‘double-cycle’ strategy, and is even more relevant to the fulfilment of the goal of common prosperity.

China’s manufacturing industry faces systemic risks in the context of global industrial chain reconstruction. At present, the industrial chain has the vulnerability of “both ends being outside”, with the degree of external dependence on key core technologies reaching more than 35 per cent, and the degree of integration between productive service industries and manufacturing industries being only 60 per cent of the level of developed countries [5]. Through the study of upstream and downstream industrial chain synergy, cross-industry technology diffusion, regional industrial gradient transfer and other coordination mechanisms, can enhance the resilience of the industrial chain [6]. Promote the transformation of “Made in China” to “intellectual creation in China”. This is of strategic value for breaking through the ‘medium technology trap’ and achieving new industrialisation.

The energy consumption of the manufacturing industry accounts for 55 per cent of the country’s total energy consumption, and the carbon emission intensity per unit of GDP is 2.3 times higher than the average level of OECD countries. Facing the challenges of international rules such as the EU carbon border adjustment mechanism, we will study the green manufacturing technology path, circular economy model and ESG evaluation system [7]. It is not only related to the fulfilment of the 2060 carbon neutral commitment, but will also promote the global value chain status to the two ends of the “smile curve”. According to UNIDO data, every 1 per cent increase in green total factor productivity can create about 1.2 million high-quality jobs. This is of dual significance in terms of breaking resource and environmental constraints and fostering new productivity.

The main innovations of this paper are as follows: (1) This paper will establish a research framework that integrates the concepts of balanced, coordinated, and sustainable development of the manufacturing industry. The connotation of these concepts will be scientifically defined based on the actual situation of the manufacturing industry’s development, as well as relevant policies and literature. (2) Different measurement methods will be employed to assess the level of balanced, coordinated, and sustainable development of the manufacturing industry. This will help clarify its spatial-temporal characteristics and evolutionary patterns.(3) Based on the findings and conclusions, targeted suggestions will be proposed to promote the balanced, coordinated, and sustainable development of the manufacturing industry. These recommendations will provide insights for achieving high-quality development in this sector.

## 2. Literature review

The government report report highlights the importance of promoting balanced, coordinated, and sustainable economic development, implementing integrated development strategies, building a modern industrial system, and pursuing high-quality development to achieve common prosperity. The manufacturing industry, as the backbone of the national economy, plays a pivotal role in realizing high-quality development and common prosperity.

Recent scholarly research has focused on the regional characteristics of the manufacturing industry in China, revealing significant patterns that are likely to persist. The overall development of the manufacturing industry exhibits a changing trend of “southwest-northeast-northwest” in terms of its center of gravity [8]. There is a notable phenomenon of homogeneous agglomeration and regional imbalance [9], with the level of disparity within developed regions being lower than that in less developed regions. Additionally, the disparity between the eastern and western regions is greater than that between the northern and southern regions [10]. Research indicates that the spatial characteristics of China’s manufacturing industry have undergone temporal changes. Initially, from 1980, the industry exhibited a south-to-east distribution pattern. However, since 2003, a north-to-west trend has emerged, with a significant shift of manufacturing activities from higher economic regions to lower-level ones. Nonetheless, certain central and western regions possess a solid industrial foundation [11]. This regional evolution demonstrates industry heterogeneity, as export-processing and labor-intensive industries concentrate along the coast, while resource-dependent sectors relocate to the north, central, and western areas [12,13]. The distribution disparities in the manufacturing industry primarily stem from factors such as economic development levels, labor force availability, and transportation infrastructure [14]. Additionally, trade costs, resource endowment, agglomeration effects, and globalization influence the spatial distribution, with varying degrees of impact depending on distance [15]. Some scholars also argue that policy biases contribute significantly to the imbalanced distribution of manufacturing industries [16].

Scholars have primarily focused on the coordinated development of the manufacturing industry, particularly the analysis of the interplay between the manufacturing industry and other sectors [17]. Research has found a dynamic synergistic relationship between the manufacturing industry and the productive service industry, as they interact and develop together [18]. However, the overall level of coordination between the manufacturing industry and the logistics industry remains relatively low, despite a growing integration trend [19]. On the other hand, a higher level of coordination between the manufacturing industry and the real estate industry has been shown to have a positive impact on total factor productivity and economic growth rate [20,21]. The integration between manufacturing and other industries plays a crucial role in achieving high-quality development. In terms of sustainable development, scholars have primarily focused on measuring the level of sustainable development [22]. Some have constructed DEA indicators based on input-output efficiency [23], while others have adopted the Sustainable Development Goals (SDGs) framework to establish comprehensive evaluation indicators. These approaches provide a more objective assessment of each city’s ability to achieve sustainable development [24–26].

## 3. Theoretical analysis

The theoretical foundation of balanced development is rooted in the innovation of coupling regional economics and new structural economics. The dynamic game of polarisation effect and diffusion effect revealed by Muldaur’s “cyclic cumulative causality theory” explains the inherent mechanism of regional imbalance in manufacturing industry. Justin Yifu Lin’s new structural economics emphasises the appropriateness of factor endowment structure and industrial upgrading, and provides theoretical support for cracking the “low-end lock” in central and western China. China’s practice has creatively developed the theoretical framework of “National Unified Big Market”, which breaks down administrative barriers through factor market reform and promotes industrial gradient transfer and spatial rebalancing. At present, there is a greater need to integrate complex systems theory and build a balanced and coordinated development model for the manufacturing industry that includes digital elements, so as to explain how the industrial Internet platform can reconfigure the geographic pattern of the manufacturing industry.

The theoretical lineage of coordinated development originated from the idea of balance between the two major categories of Marx’s theory of social reproduction. In the context of industrial chain modernisation, it has evolved into the theory of “chain cluster synergy”. The intersection of Porter’s theory of industrial clusters and Qian Xuesen’s system engineering has formed the analytical paradigm of ‘three-chain integration’ of industrial chain, innovation chain and value chain. Aiming at the characteristics of China’s manufacturing industry as “big but not strong”, academics have put forward the theory of “chain modernisation”, which emphasises the construction of ecosystem led by core enterprises and collective breakthroughs in “necked” technologies. Mechanism. The new development stage needs to introduce the theory of complex adaptive system to analyse the cross-domain synergistic law of data elements and traditional production factors in the project of “East counts, West counts”. The new development stage needs to introduce the theory of complex adaptive systems to analyse the cross-domain synergy between data factors and traditional production factors in the “East Meets West” project, as well as the nested evolution paths of the global value chain and the domestic value chain in the context of the “double cycle”.

The theoretical innovation of sustainable development is reflected in the in-depth integration of the idea of ecological civilisation and the theory of new industrialisation. The environmental Kuznets curve theory has been expanded into a two-way driving model of “industrial upgrading and green transformation” in the Chinese context. The Peres technology-economy paradigm theory provides a new perspective for understanding the coupling of the green technology revolution and the transformation of the manufacturing industry. The theory of circular economy has been sublimated into the theory of industrial metabolism of “waste-free city” through China’s practice, which emphasises the systematic optimisation of material, energy and information flows. Currently, it is urgent to integrate the law of industrial leap under the goal of “dual carbon”, and build a new theoretical framework that includes the mechanism of ESG value endogenous, and the digital twin technology empowering green manufacturing. In order to reveal the multi-scale transmission effect of green total factor productivity enhancement.

## 4. Materials and methods

Analyzing the characteristics and explaining the connotation of balanced, coordinated, and sustainable development of the manufacturing industry is crucial for understanding the intrinsic relationship between the current state of manufacturing industry development and the society and economy. This understanding is essential for achieving high-quality development. In this section, we will define the connotation of the three aspects based on economic development theories, policy literature, and the current situation of manufacturing industry development.

### 4.1 Balanced development of the manufacturing industry

Various theories on non-equilibrium development of regional economies, such as the principle of cyclic accumulation causality, growth pole theory, and regional economic gradient transfer theory, have been proposed by Western economists. These theories suggest that while a non-equilibrium strategy can promote economic progress to some extent, significant deviations from the equilibrium state are challenging to regulate through market mechanisms. Moreover, the scale effect exacerbates this non-equilibrium state, leading to potential durability issues in economic development [27]. China, influenced by differences in natural resource endowment and uneven allocation of social resources across regions, has long been guided by the theory of unbalanced development. Undoubtedly, the strategy of non-equilibrium development has significantly improved China’s economic development level. However, it has also resulted in challenges, such as widening regional development gaps, deepening social inequalities between the rich and the poor, and hindering long-term socio-economic development. Therefore, achieving balanced regional development is an imperative choice for China’s development in the current situation [28]. The Government emphasises that that the strategy of balanced development has become a crucial economic development strategy in China, with a focus on regional balance. The Government stated that the main contradiction in Chinese society has shifted to the contradiction between the growing needs of the people for a better life and the unbalanced and inadequate development. Solving the problem of unbalanced development has become an urgent matter. It is evident that the strategy of balanced development serves as a corrective measure and solution to address regional disparities in China, which is vital for the country’s economic development.

The theory of regional equilibrium, which underpins the concept of balanced development, refers to achieving balanced development within and between regions. It involves the inter-regional mobility of production factors and aims to converge the level of economic development among regions. This theory advocates for a balanced deployment of productive forces within regions through macroeconomic control measures, spatially balanced investment, and the realization of balanced development in regional economies [29]. Based on this, this paper proposes that balanced development in the manufacturing industry refers to a shrinking development gap among manufacturing industry sectors in the region. In other words, it signifies a trend where the development gap in the manufacturing industry becomes smaller and smaller. It should be noted that balanced development in the manufacturing industry does not imply achieving an average or equal development level across all regions. Instead, it aims to achieve relatively balanced development that ensures Pareto improvement and equitable development for local interests, without sacrificing the advanced regions’ manufacturing industry for the sake of developing backward regions.

To analyze the regional equilibrium of the manufacturing sector, this paper focuses on the differences in the development levels of the manufacturing industry across regions. Traditional research methods do not adequately address the measurement of the manufacturing industry’s equilibrium level. Therefore, this paper proposes the use of relative indicators based on the output value and the number of employees in each sector of the manufacturing industry to measure the regional equilibrium level. By calculating the development level of the manufacturing industry in each region and the relative difference between the highest level of development in a region, the measurement of the development gap and equilibrium level of the manufacturing industry can be obtained. This approach provides an intuitive and scientific calculation of the gap in the regional development of the manufacturing industry, revealing the characteristics of regional differences. Moreover, it enables a specific measurement of the development level differences among various industrial sectors within the manufacturing industry. This approach aligns with the definition of balanced development of the manufacturing industry in this paper. The specific indicators for measuring the balanced development level of the manufacturing industry are shown in formula (1):


$$Y_{ijt}=\left(M_{ijt}/L_{ijt}\right)/(M_{sjt}/L_{sjt})$$ (1)

Let *M*_*ijt*_ represent the output value of industry j in province *i* in year *t*, *L*_*ijt*_ represent the number of people employed in industry *j* in province *i* in year *t*, *M*_*sjt*_ represent the highest output value of industry *j* in province *s* in year *t*, and *L*_*sjt*_ represent the number of people employed in industry *j* in province *s* in year *t*. The per capita output value of the industry is used as a measure of the relative development gap at the industry level. A higher value indicates a higher level of equilibrium in the manufacturing industry, while a lower value indicates a larger relative gap.

### 4.2 Coordinated development of the manufacturing industry

The Government’s report states that coordinated development develops in appropriate proportions. Coordinated development is of great significance to economic development in the new era, and the concept of coordinated development should be adhered to. Scholars have also provided definitions of coordinated development. They highlighted the importance of the relationship of coordination between elements and the need to prevent polarization and vicious cycles [30,31]. While others emphasized respecting objective laws and using scientific methods to promote cooperation and a virtuous cycle among elements [32,33]. It is evident that coordinated development requires addressing the unity of opposites and effectively managing major relationships. In the context of the manufacturing industry, coordinated development is reflected in the interplay between manufacturing input and output, as well as the coordination and matching between industrial sectors [34]. It emphasizes the relationship between factor structure and the combination of various sectors within the manufacturing industry. Therefore, the coordinated development of the manufacturing industry involves managing the input-output relationship of manufacturing factors, adjusting the proportion of manufacturing sectors, and improving the efficiency of resource allocation. This includes optimizing the factor structure relationship and achieving inter-industry organic combination. It is important to emphasize that the coordinated development of the manufacturing industry aims to achieve Pareto improvement, maximize the advantages of each industry, and enhance the efficiency of resource allocation [35]. It is a necessary requirement for the manufacturing industry to gain competitive advantage and an inevitable choice for its development.

This paper first employs total factor productivity to evaluate the development level of the manufacturing sector from the perspective of input-output efficiency, specifically the factor structure. Secondly, the paper utilizes a coupling coordination model to measure the interactive synergistic relationship among the development levels of various sectors within the manufacturing industry. This model analyzes the coupling relationship within the manufacturing industry and determines the level of coordination. The ACF method is employed to measure total factor productivity, overcoming the issue of covariance and eliminating the influence of exogenous random shocks. The coupled coordination model assigns weights to each sector within the manufacturing industry based on the contribution value of their development level to economic development. This allows for the measurement of the structural relationship between the development levels of each sector within the manufacturing industry, *i.e*., the level of coordination within the manufacturing industry. The specific measurement formula is shown in the following equation.


$$C=3\sqrt[{3}]{U_{1}\times U_{2}\times U_{3}}/(U_{1}+U_{2}+U_{3})$$ (2)

$$T=\alpha U_{1}+\beta U_{2}+\gamma U_{3}$$ (3)

$$D=\sqrt{C\times T}$$ (4)

*C* denotes the degree of coupling; *T* is the comprehensive coordination index; *D* is the degree of coupling coordination, the larger the value, the higher the degree of coordination; considering the numerous manufacturing industry segments, which is not conducive to the study, therefore, the manufacturing industry is divided into three major types of labor-intensive, capital-intensive and technology-intensive for analysis. *U*_*1*_, *U*_*2*_, *U*_*3*_ denote the total factor productivity of labor-intensive manufacturing industry, total factor productivity of capital-intensive manufacturing industry and total factor productivity of technology-intensive manufacturing industry after standardization, respectively. The weights of *α*, *β*, *γ* are to be determined to reflect the importance of labor-intensive, capital-intensive and technology-intensive manufacturing industries, and this paper takes α=0.2, β=0.3, γ=0.5 according to the importance of each manufacturing industry in the national economy. The level of harmonization is shown in Table 1.

**Table 1:** Criteria for the evaluation of the level of harmonization.

Coordination level	coherence	Coordination level	coherence
Extreme Dissonance	(0, 0.1]	Extreme Dissonance	(0.5, 0.6]
Severe Dissonance	(0.1, 0.2]	Severe Dissonance	(0.6, 0.7]
Moderate Dissonance	(0.2, 0.3]	Moderate Dissonance	(0.7, 0,8]
Mildly dysfunctional	(0.3, 0.4]	Mildly dysfunctional	(0.8, 0.9]
Nearly dysfunctional	(0.4, 0.5]	Nearly dysfunctional	(0.9, 1]

### 4.3 Sustainable development of the manufacturing industry

The concept of sustainable development was first put forward in the World Programme for the Conservation of Nature in 1980. On this basis, our government report pointed out that the scientific concept of development is a major strategic idea, pointing out that sustainable development is one of the basic requirements for economic and social development, and since then, taking the road of sustainable development has become the most important way of economic development in China.The 2015 United Nations Development Summit pointed out that the sustainable development goals refer to the complete resolution of social, economic and ecological three-dimensional development problems from 2015 to 2030, and the achievement of long-term healthy development. development problems and achieve long-term healthy development. Innovation is the first driving force of economic development, and it is necessary to implement the strategy of innovative development, while promoting the level of green development, promoting harmony between human beings and nature, and taking the road of sustainable development. It can be seen that sustainable development is to achieve social, economic and ecological co-development, emphasising the durability of development.

The essence of manufacturing development is a resource-based production process, and resources are limited, to achieve long-term development of the manufacturing industry, we must break through the resource constraints. Innovation, as the fundamental driving force of modernisation, is the core force of all economic development and the inevitable choice for the long-lasting development of the manufacturing industry [36]. Therefore, this paper proposes that the sustainable development of the manufacturing industry refers to the healthy development mode that takes innovation as the driving force and resources as the basis to achieve the common development of social, economic and ecological goals. Specifically manifested in three aspects, first, the development of manufacturing industry should play an important supporting role for social and economic development. Secondly, the manufacturing industry is growing under the impetus of technological progress. Third, in the resources, environmental constraints in the manufacturing industry to achieve long-term development. The sustainable development of the manufacturing industry is the potential for future development and the ability of the manufacturing industry to develop in a favourable manner, and is based on the premise of ensuring the sustainable development of resources and the environment, and taking innovation as the driving force to achieve the development of social, economic and ecological benefits..

#### 4.3.1 Manufacturing sustainable development index construction.

Based on the previous analysis, sustainable development of the manufacturing industry is a healthy way to achieve economic, social, and ecological goals, with innovation as the driving force and resource utilization as the foundation. It is evident that sustainable development of the manufacturing industry encompasses various aspects, and a single economic indicator cannot fully capture its rich connotation. To measure the level of sustainable development of the manufacturing industry, this paper employs a comprehensive evaluation index. The overall goal of sustainable development of the manufacturing industry is to achieve common development of society, economy, and ecology, emphasizing comprehensive and persistent development [5]. As the essence of the manufacturing industry is to conduct production activities based on limited resources, improving resource utilization efficiency is necessary for its long-term development. Innovation, as the fundamental driving force of modernization, is the core force of all economic development and an inevitable choice for the long-term development of the manufacturing industry. Therefore, this paper constructs an evaluation index of sustainable development of the manufacturing industry based on the overall goal of manufacturing industry development, innovation level, and resource utilization in three dimensions.

(1) Overarching goals. The United Nations sustainable development goals aim to address social, economic, and ecological issues. For China’s manufacturing industry, sustainable development requires moderate and stable growth as the basic economic goal, improving people’s quality of life as the social goal, and pursuing ecological benefits as the environmental goal. This paper constructs an evaluation index for the general objective of sustainable development of the manufacturing industry based on economic, social, and ecological benefits. (2) Innovation level. Innovation is the fundamental driving force of economic development. However, China’s manufacturing industry lacks core competitiveness and innovation input, and innovation output efficiency is low. This paper measures the level of innovation in sustainable development of the manufacturing industry based on innovation input and output. (3) The rapid development of China’s manufacturing industry has relied on demographic and natural resources, but with their gradual depletion, the structural shortage of resources has become a pressing issue. To achieve sustainable development, it is necessary to improve resource utilization efficiency, focusing on capital, labor, and energy allocation. This paper measures the resource level of sustainable development of the manufacturing industry based on these three dimensions. Specific indicators are constructed as shown in Table 2.

**Table 2:** Evaluation indicator system for sustainable development in manufacturing.

Overall Objectives	Economic Benefits	Profitability
		Production value contribution rate
	Social Benefits	Tax contribution rate
		Employment Contribution Rate
	Ecological Benefits	Green governance capacity
		Environmental Protection Capability
Innovation Level	Innovation Input	Intensity of R&D Personnel Input
		R&D Expenditure Investment Intensity
	Innovation Output	Patents per capita
		Technology Market Turnover per Capita
		New Product Sales Revenue Rate
Resource Utilization	Capital Allocation	Capital productivity
	Labor allocation	Labor productivity
	Energy allocation	Energy productivity

#### 4.3.2 Methodology for measuring the level of sustainable development.

This paper uses the entropy weight method to comprehensively evaluate the level of sustainable development of the manufacturing industry. The methodology allows for a more rational and objective comprehensive evaluation or ranking. The basic steps are as follows.

(1) Applying the method of polar deviation to eliminate inconsistencies in the attributes and magnitudes of the indicators, all indicators are transformed into positive indicators to obtain a normalization matrix$\left\{X_{jt}\right\}$.

$$\left(2\right)\text{Positive indicators}:X_{jt}^{+}=\frac{x_{jt}-\min x_{j}}{\max x_{j}-\min x_{j}}$$ (5)

$$\text{Negative indicators:}X_{jt}^{-}=\frac{\max x_{j}-x_{jt}}{\max x_{j}-\min x_{j}}$$ (6)

*j* denotes the measure and t denotes time.

(3) Calculate information entropy$E_{j}$and$W_{j}$.

$$E_{j}=\ln\frac{1}{T}*\sum_{t=1}^{T}P_{jt}*\ln P_{jt},{\text{P}}_{\text{jt}}=X_{\text{jt}}/\sum_{t=1}^{T}X_{jt},W_{j}=(1-E_{j})/\sum_{j=1}^{J}\left(1-E_{j}\right)$$ (7)

(4) Construction of a weighting matrix for indicators to measure the level of sustainable development$R_{jt}$

$$R_{jt}=W_{j}*X_{jt}.$$ (8)

(5) Determination of optimal $Q_{j}^{+}$ and least optimal solutions $Q_{j}^{-}$ based on a weighting matrix *R*.

$$\{\begin{array}{l} Q_{j}^{+}=(\max R_{1t},\max R_{2t},L,\max R_{jt})\\ Q_{j}^{-}=(\min R_{1t},\min R_{2t},L,\min R_{jt}) \end{array}$$ (9)

(6) Calculate the Euclidean distance$D_{t}^{+}$ and$D_{t}^{-}$.

$$\{\begin{array}{l} D_{t}^{+}=\sqrt{\sum_{j=1}^{J}{\left(Q_{j}^{+}-R_{jt}\right)}^{2}}\\ D_{t}^{-}=\sqrt{\sum_{j=1}^{J}{\left(Q_{j}^{-}-R_{jt}\right)}^{2}} \end{array}$$ (10)

(7) Calculation of the level of sustainable development$C_{t}$.

$$C_{t}=\frac{D_{t}^{-}}{D_{t}^{+}+D_{t}^{-}}$$ (11)

### 4.4 Data description

The research covers 30 provinces in China from 2011 to 2020, excluding Tibet, Hong Kong, Macao, and Taiwan due to missing data. The 30 sub-industries of the manufacturing industry are also included, with the exception of rubber, plastics, automobile, railroad, ship, and aerospace transportation equipment manufacturing industries, which are combined for analysis. Data is obtained from various sources, including China Statistical Yearbook, China Industrial Statistical Yearbook, provincial statistical yearbooks, the official website of the National Bureau of Statistics, EPS Global Statistical Data Platform, and GuotaiAn database. The study excludes ST and ST* listed enterprises and adjusts nominal variables using 2011 as the base period. Operating income is deflated by the factory price index of the enterprise’s location, while net fixed assets and intermediate input indicators are deflated by the price index of investment in fixed assets and the consumer price index, respectively. The study fills in missing values using the interpolation method and estimates manufacturing industry data not publicized in the database using the ratio of total assets of regulated industrial enterprises to the total assets of manufacturing enterprises.

## 5. Results

### 5.1 Analysis of the balanced development

To facilitate the analysis, the manufacturing industry is divided into 30 sectors based on industry codes, and the country is divided into the East, Central, West, and Northeast regions based on geographic location. Balanced indicators established for the manufacturing industry are used to calculate the balanced level of development for each two-digit industry code in the country and major regions of China. The specific results of these measurements are summarized in Table 3.

**Table 3:** Equilibrium level of manufacturing sub-sectors.

Manufacturing double-digit industries	Balance	
	Twelfth Five	Thirteenth Five
Agri-food processing industry	0.4503	0.1811
Food Manufacturing	0.5032	0.4949
Beverage Manufacturing	0.3201	0.3065
Tobacco Products	0.2171	0.2264
Textile Industry	0.4061	0.4144
Textile clothing, shoes, hats manufacturing	0.4871	0.4791
Leather, Fur, Feather and Products	0.2188	0.2569
Wood processing and wood, bamboo, rattan, palm and grass products industry	0.4540	0.3674
Furniture Manufacturing	0.4060	0.4074
Paper and paper products industry	0.3996	0.2879
Printing and recording media reproduction	0.3019	0.3080
Arts, Education and Sporting Goods Manufacturing	0.2976	0.2708
Petroleum Processing, Coking and Nuclear Fuel Processing Industry	0.1824	0.2394
Chemical raw materials and chemical products manufacturing	0.3429	0.2767
Pharmaceutical Manufacturing	0.6216	0.5161
Chemical Fiber Manufacturing	0.2590	0.3272
Rubber and plastic products industry	0.5351	0.5880
Non-metallic mineral products industry	0.4255	0.5285
Ferrous metal smelting and rolling processing industry	0.2816	0.3568
Non-ferrous metal smelting and rolling processing industry	0.4218	0.5139
Metal Products Industry	0.3890	0.5614
General Equipment Manufacturing	0.5554	0.5276
Specialty Equipment Manufacturing	0.5471	0.3987
Transportation	0.3012	0.4082
Electrical machinery and equipment manufacturing	0.5205	0.5886
Communication equipment, computer and other electronic equipment manufacturing	0.4092	0.4580
Instrumentation Manufacturing	0.2653	0.4209
Other Manufacturing Industries	0.3032	0.1346
Waste Resources and Waste Materials Recycling and Processing Industry	0.3396	0.3810
Metal products, machinery and equipment repair industry	0.1999	0.2345

The results in Table 4 indicate that the majority of manufacturing industries at the national and regional levels have shown an upward trend in their balanced level. The industries with representative growth include petroleum processing, coking and nuclear fuel processing, chemical fiber manufacturing, ferrous metal smelting and rolling, non-ferrous metal smelting and rolling, metal products, transportation, and instrumentation manufacturing, comprising both capital-intensive and technology-intensive industries. The latter have shown a wide range of growth in China’s manufacturing industry balance level, indicating a shrinking regional development gap. This is largely due to the national coordinated development strategy, which has continuously improved the manufacturing industry’s regional balance level. However, some industries, such as textiles, furniture manufacturing, leather, fur, feather and its products, and non-metallic mineral products, have shown a declining trend in their balanced level in the northeast, while the rest of the country and other regions have shown an upward trend. This suggests that the development gap between the northeast and other regions is widening, possibly due to the northeast’s reliance on its industrial base during the twelfth five-year plan period, resulting in higher development levels.

**Table 4:** Balanced level of manufacturing sub-sectors.

East		Central		Northeast		West	
Twelfth Five	Thirteenth Five	Twelfth Five	Thirteenth Five	Twelfth Five	Thirteenth Five	Twelfth Five	Thirteenth Five
0.4637	0.2235	0.4671	0.1792	0.5939	0.1685	0.3899	0.1471
0.5049	0.4955	0.4679	0.4815	0.6898	0.5154	0.4700	0.4959
0.3044	0.2538	0.2972	0.2960	0.3634	0.3076	0.3349	0.3598
0.2805	0.2932	0.1630	0.2027	0.1225	0.1256	0.2149	0.2062
0.4250	0.4506	0.4057	0.4122	0.3183	0.2258	0.4131	0.4343
0.4624	0.4719	0.5251	0.5636	0.8384	0.4397	0.3929	0.4504
0.1519	0.2167	0.2359	0.2550	0.5858	0.5378	0.1701	0.2178
0.4137	0.3464	0.4544	0.4813	0.6807	0.3324	0.4286	0.3338
0.3602	0.4243	0.5091	0.5145	0.5508	0.2765	0.3521	0.3693
0.4723	0.3351	0.4007	0.2932	0.4182	0.2002	0.3277	0.2658
0.2662	0.2743	0.3380	0.3393	0.3480	0.2493	0.3020	0.3376
0.2696	0.3157	0.2653	0.2666	0.3975	0.1599	0.3135	0.2624
0.3281	0.3929	0.1199	0.1846	0.0880	0.1272	0.1098	0.1603
0.5029	0.3845	0.2603	0.2121	0.3738	0.2669	0.2342	0.2166
0.6635	0.5512	0.5823	0.4950	0.6579	0.5468	0.5952	0.4873
0.3270	0.4001	0.2128	0.2912	0.1769	0.2291	0.2447	0.3074
0.4885	0.5269	0.6106	0.6447	0.6716	0.6177	0.4991	0.6046
0.4317	0.5588	0.3813	0.4852	0.6102	0.4794	0.3936	0.5380
0.3885	0.4796	0.2327	0.3158	0.1996	0.2553	0.2333	0.2952
0.4519	0.5286	0.5007	0.5754	0.2748	0.2907	0.3916	0.5280
0.4017	0.5293	0.3793	0.5847	0.4193	0.5602	0.3746	0.5783
0.5894	0.5286	0.6173	0.6063	0.6830	0.4698	0.4560	0.4995
0.5278	0.3653	0.5860	0.4361	0.7227	0.3269	0.4955	0.4282
0.3591	0.4651	0.2300	0.3852	0.3191	0.4912	0.2825	0.3464
0.4692	0.5496	0.4970	0.5991	0.5349	0.4043	0.5760	0.6687
0.4725	0.4961	0.3363	0.4180	0.3610	0.3428	0.4047	0.4766
0.2511	0.4020	0.2633	0.5244	0.2429	0.2887	0.2853	0.4177
0.2541	0.1409	0.3009	0.1258	0.4322	0.1096	0.3139	0.1405
0.3648	0.3779	0.3870	0.4507	0.2604	0.3903	0.3123	0.3434
0.2456	0.2721	0.2511	0.3540	0.1732	0.1846	0.1376	0.1488

However, the transformation and upgrading of the manufacturing industry, as well as geographic location, have affected the development level of the manufacturing industry in the northeast, resulting in a widening development gap with other regions. Additionally, some industries, such as agricultural and food processing, wood processing and wood, bamboo, rattan, palm, grass products, paper and paper products, and chemical raw materials and chemical products manufacturing, have shown a declining trend in their balanced level, indicating an expanding development gap. These are mainly resource-intensive industries, with development affected by resource distribution and a high degree of agglomeration, resulting in a decline in the level of regional equilibrium. The industries with a lower overall balanced level include tobacco products, petroleum processing, coking and nuclear fuel processing, metal products, and machinery and equipment repair, which are mainly labor- and capital-intensive industries. The development of the manufacturing industry in these industries is more varied, subject to the development of the basis and the level of regional economic development, resulting in a lower regional balance level. Notably, the eastern region has a higher overall balanced level than other regions, as it is the main region for the distribution of manufacturing industries in China, with a high density of manufacturing industries and a high level of regional integration.

### 5.2 Analysis of the coordinated development

#### 5.2.1 Manufacturing factor structure level.

Table 5 presents the factor structure level of the manufacturing industry, focusing on regional distribution. The highest development level of technology-intensive industries is observed in the east, with a value of 7.1413. Capital-intensive industries in the central, northeastern, and western regions also exhibit relatively high development levels, with values of 7.2300, 6.8019, and 6.9088, respectively. On the other hand, the western region shows a lower factor structure level for technology-intensive industries, with a value of 6.5284, indicating a relatively lower overall factor structure level for the manufacturing industry in this region. Analyzing the growth rate of the factor structure for each industry, labor-intensive industries in the eastern region demonstrate the fastest growth rate at 0.5286. The central region exhibits the highest growth rate for capital-intensive industries at 0.4817, while the northeastern region shows the highest growth rate for labor-intensive industries at 1.5974. The western region, on the other hand, displays the highest growth rate for capital-intensive industries at 0.4615. The rapid growth of labor-intensive industries in the eastern region can be attributed to recent population agglomeration, as the region has experienced a significant influx of labor factors. This influx has objectively enhanced the level of labor input in the eastern region. The growth rate of capital-intensive industries in the central and western regions is mainly influenced by China’s industrial restructuring and upgrading. The transformation and upgrading of the manufacturing industry, which requires capital support, has resulted in the rapid development of capital-intensive industries in these regions. Overall, manufacturing industries in all regions are transitioning from labor and capital-intensive industries to capital and technology-intensive industries, leading to continuous optimization of the factor structure of the manufacturing industry. It is important to note that although the growth rate of labor-intensive industries in the eastern region is the highest, it still has the lowest development level in the region. As the leader of China’s manufacturing industry, the eastern region has always been dominated by capital- and technology-intensive industries, which exhibit high development efficiency and a high factor structure level.

**Table 5:** Level of manufacturing factor structure.

Region	Industry	2011	2012	2013	2014	2015	2016	2017	2018	2019	2020	均值
East	Labor	6.2549	6.3084	6.4335	6.4264	6.3987	6.3534	6.4330	6.5107	6.5482	6.7835	6.4451
	Capital	6.8165	6.8040	6.9399	6.8543	6.7870	6.8526	7.0682	7.1039	7.1057	7.1005	6.9433
	Technology	7.2607	7.2038	6.9944	7.0191	7.0328	7.0529	7.0974	7.1713	7.3215	7.2595	7.1413
Central	Labor	6.4096	6.1707	6.2341	6.2149	6.2488	6.3752	6.4533	6.6187	6.9411	6.8623	6.4529
	Capital	7.0622	7.0913	7.1305	7.1976	7.1404	7.2515	7.2228	7.3116	7.3480	7.5439	7.2300
	Technology	6.5105	6.4107	6.3492	6.3300	6.3434	6.3899	6.4301	6.4265	6.4841	6.5436	6.4218
Northeast	Labor	5.4932	6.0896	6.2744	6.9924	6.6796	6.8211	6.9121	6.8925	6.7200	7.0906	6.5965
	Capital	6.8861	6.7871	6.5987	6.7400	6.3238	6.5399	6.8934	6.9759	7.0792	7.1938	6.8018
	Technology	6.4876	6.2221	6.3313	6.3797	6.2928	6.3085	6.4257	6.3913	6.4678	7.1457	6.4453
West	Labor	6.5344	6.4875	6.5189	6.5866	6.6737	6.6871	6.7153	6.6871	6.7536	6.8267	6.6471
	Capital	6.7564	6.6706	6.8171	6.7963	6.7419	6.8816	7.0288	7.0653	7.1119	7.2179	6.9088
	Technology	6.4536	6.2355	6.3696	6.4725	6.5771	6.6171	6.6160	6.5820	6.6410	6.7197	6.5284

#### 5.2.2 Manufacturing coordination level.

**Fig 1:**
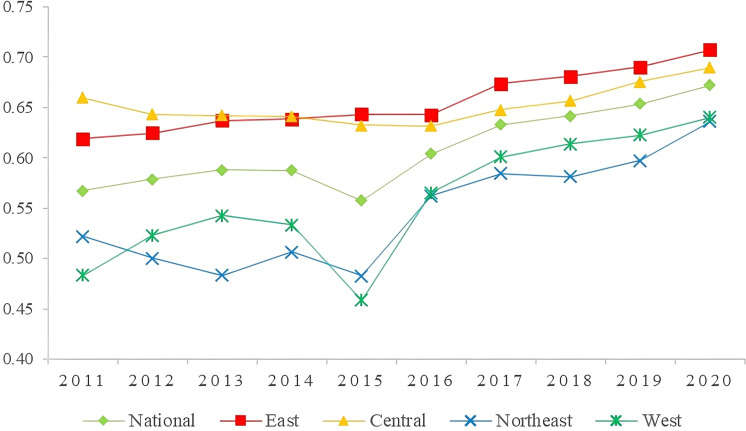
Level of subregional manufacturing coordination.

To facilitate comparative analysis across time and space, this study calculated the coordination level of the manufacturing industry in each province of China using the manufacturing coordination index for the period from 2011 to 2020. The results of this calculation are presented in Table 6. To analyze the coordination level of the manufacturing industry in different regions, China was divided into four regions based on geographic location: the east, middle, west, and northeast. The development trend of coordination in each region was analyzed, and changes in the level of coordination of the manufacturing industry in the country and the four regions from 2011 to 2020 are depicted in Fig 1.

**Table 6:** Level of manufacturing coordination.

Province	2011	2012	2013	2014	2015	2016	2017	2018	2019	2020	均值
Beijing	0.6887	0.6888	0.7061	0.7038	0.7115	0.7300	0.7484	0.7566	0.7723	0.7764	0.7283
Tianjin	0.3001	0.4249	0.4545	0.4976	0.5424	0.4366	0.5428	0.5692	0.5705	0.6837	0.5022
Hebei	0.6580	0.6659	0.6794	0.6726	0.6884	0.7122	0.7127	0.7147	0.7067	0.7077	0.6919
Shanxi	0.6390	0.6215	0.6168	0.5953	0.5906	0.5463	0.5829	0.5726	0.5995	0.6223	0.5987
Neimeng	0.5955	0.5479	0.5681	0.5222	0.4847	0.6068	0.6384	0.6476	0.6497	0.6578	0.5919
Liaoning	0.5774	0.5668	0.5244	0.5657	0.5408	0.5508	0.6129	0.6285	0.6387	0.6518	0.5858
Jilin	0.5305	0.4612	0.4561	0.4483	0.4653	0.5243	0.5677	0.5310	0.5525	0.6103	0.5147
Heilongjiang	0.4588	0.4730	0.4690	0.5058	0.4423	0.6127	0.5738	0.5853	0.6013	0.6461	0.5368
Shanghai	0.8989	0.9311	0.8778	0.8757	0.8683	0.8676	0.8794	0.8780	0.9312	0.9157	0.8924
Jiangsu	0.6077	0.5922	0.5959	0.5740	0.5752	0.5835	0.6146	0.6400	0.6474	0.6573	0.6088
Zhejiang	0.5715	0.5617	0.5790	0.5806	0.5925	0.6127	0.6366	0.6476	0.6525	0.6677	0.6102
Anhui	0.6991	0.6641	0.6840	0.6781	0.6904	0.7147	0.7024	0.7069	0.7185	0.7182	0.6976
Fujian	0.6370	0.5910	0.5967	0.5866	0.5781	0.5724	0.6209	0.6368	0.6572	0.6634	0.6140
Jiangxi	0.6879	0.6742	0.6966	0.7200	0.7146	0.7200	0.7225	0.7095	0.7221	0.7532	0.7121
Shandong	0.6490	0.6448	0.6631	0.6555	0.6542	0.6812	0.7284	0.7360	0.7480	0.7702	0.6930
Henan	0.6220	0.6056	0.6081	0.5916	0.5974	0.6333	0.6201	0.6379	0.6469	0.6445	0.6208
Hubei	0.6272	0.6252	0.6043	0.6475	0.6324	0.5967	0.6691	0.6807	0.6843	0.6789	0.6446
Hunan	0.6854	0.6708	0.6460	0.6159	0.5689	0.5824	0.5920	0.6346	0.6847	0.7215	0.6402
Guangdong	0.6684	0.6426	0.6540	0.6731	0.6581	0.6563	0.6909	0.7257	0.7236	0.7281	0.6821
Guangxi	0.6771	0.6277	0.6477	0.6446	0.5370	0.5768	0.6458	0.7042	0.7022	0.7002	0.6463
Hainan	0.5098	0.5038	0.5661	0.5678	0.5664	0.5783	0.5663	0.5065	0.4969	0.5055	0.5367
Chongqing	0.6151	0.5875	0.5660	0.5569	0.5754	0.5983	0.6352	0.6530	0.6584	0.6984	0.6144
Sichuan	0.6846	0.6286	0.6579	0.6520	0.6692	0.6589	0.6785	0.6886	0.7064	0.7115	0.6736
Guizhou	0.4113	0.4166	0.4523	0.4767	0.4714	0.4819	0.5047	0.4878	0.4893	0.5253	0.4717
Yunnan	0.6399	0.5966	0.6314	0.4402	0.5879	0.7356	0.7368	0.7374	0.7512	0.7648	0.6622
Shannxi	0.4611	0.4293	0.4323	0.5375	0.5334	0.5375	0.5764	0.5738	0.5777	0.6196	0.5278
Gansu	0.4178	0.4401	0.4797	0.4893	0.5127	0.5650	0.5690	0.5841	0.5486	0.5375	0.5144
Qinghai	0.5661	0.5419	0.5533	0.5466	0.5427	0.5554	0.5424	0.5589	0.5928	0.6135	0.5614
Ningxia	0.2872	0.3094	0.3296	0.3245	0.2440	0.1634	0.3568	0.3848	0.4359	0.4412	0.3277
Xinjiang	0.6646	0.6278	0.6533	0.6760	0.7197	0.7392	0.7301	0.7366	0.7395	0.7719	0.7059

From 2011 to 2020, the level of coordination in the manufacturing industry in most provinces in China exhibited an overall upward trend, as shown in Table 6. However, the rate of increase in coordination level varied across provinces, with Shanxi and Hainan experiencing a decline. Fig 1 further illustrates that the eastern coastal and central regions consistently maintained a level of coordination on par with the national average, with the eastern coastal region leading the way. In recent years, some provinces in the western region have also shown a clear upward trend in the level of coordination.

The level of manufacturing coordination in the eastern region increased from 0.6198 in 2011 to 0.7076 in 2020, transitioning from primary coordination to intermediate coordination. Among the four major regions, the eastern region achieved the highest level of manufacturing coordination. This is exemplified by Shanghai and Beijing, which ranked first and second in the country with average coordination values of 0.8924 and 0.7283, respectively. The eastern region, mostly situated along the coast, benefits from advantageous geographic location, developed transportation infrastructure, and a concentration of talent. These factors contribute to the high level of manufacturing development in the region, particularly in technology-intensive industries. However, the development level of labor-intensive industries in the eastern region is relatively low, which poses a constraint on the overall coordination level of the manufacturing industry. This is primarily due to the impact of industrial transfer, with traditional labor-intensive industries relocating to the central and western regions, thereby reducing their own development level. Additionally, labor-intensive industries are also affected by the ongoing transformation and upgrading of the manufacturing industry, resulting in their relatively lower level of development.

From 2011 to 2020, the coordination level of the manufacturing industry in the central and northeastern regions of China remained at a primary level. Although there was an increase in coordination, the growth trend was not significant. This indicates that the manufacturing industry in these regions is facing challenges and urgently needs transformation and upgrading to change the current situation. The northeastern region, being China’s industrial base, is primarily composed of traditional manufacturing industries with low efficiency and a single industrial structure, resulting in a low level of coordination. Similarly, the central region, being the recipient of industries from both the eastern and western regions, faces challenges in the development of manufacturing industries. On one hand, it is impacted by the siphoning effect of the eastern region, leading to insufficient development of technology-intensive industries. On the other hand, it faces competition from the western region in labor-intensive industries, resulting in a relatively low level of development. In terms of specific sub-indicators, the central region is dominated by capital-intensive industries, which contributes to its higher development level compared to other regions. However, the average value of the three indicators of manufacturing industry coordination level in the northeastern region is at a low level, significantly restricting the overall coordination level. Notably, Jilin Province has the lowest level of coordination in the country, with a coordination value of 0.5147. Therefore, it is crucial for the central and northeastern regions to enhance the input-output efficiency of the manufacturing industry, promote the upgrading of the industrial structure, and improve the coordination level of the manufacturing industry.

The coordination level of the manufacturing industry in the western region has shown significant improvement from 2011 to 2020, with the coordination value increasing from 0.4831 to 0.6401. This transition from near-dislocation to primary coordination is the most notable progress among the four major regions. The main driving factors behind this improvement are twofold. Firstly, the western region has become a major destination for the transfer of China’s manufacturing industry, resulting in a substantial inflow of manufacturing enterprises. This has positively impacted the diversity and development level of the manufacturing industry in the region. Secondly, the implementation of economic strategies such as the Belt and Road Initiative has created more opportunities for the development of the manufacturing industry in the western region. This has facilitated the upgrading of the industrial structure, leading to a higher level of coordination in the manufacturing industry. It is worth mentioning that certain provinces in the western region have achieved a high level of manufacturing coordination. For instance, Sichuan, Yunnan, and Xinjiang have coordination values of 0.6736, 0.6622, and 0.7059, respectively. These provinces possess a strong manufacturing foundation and significant advantages in the industry. Particularly, Sichuan has a higher level of technology-intensive industries. Conversely, provinces such as Ningxia, Gansu, and Guizhou have relatively low levels of manufacturing coordination, ranking near the bottom in the country with coordination values of 0.3277, 0.5144, and 0.4717, respectively. This is mainly attributed to the weak manufacturing foundation, unfavorable geographical conditions, and low economic development level in these provinces, which significantly hinder the coordinated development of the manufacturing industry.

### 5.3 Analysis of the sustainable development

The level of sustainable development in China’s manufacturing industry has shown an overall upward trend from 2011 to 2020, as indicated in Table 7. The sustainable development level increased from 0.2815 in 2011 to 0.3230 in 2020, representing a growth of 4.15%. This upward trend demonstrates the continuous progress of China’s manufacturing industry in terms of sustainable development. Analyzing the specific sub-indicators, the overall goal of sustainable development in the manufacturing industry has experienced a downward trend. This can be attributed to the decline in the goal of economic efficiency within the industry. With the transformation and development of the economy, the proportion of China’s real economy has decreased while the virtual economy and service industry have grown rapidly. This phenomenon of de-industrialization has led to a decline in the economic efficiency of the manufacturing industry. On the other hand, the manufacturing sustainable development innovation index indicates a clear growth trend in China’s manufacturing technology innovation level. The index increased from 0.1326 in 2011 to 0.2601 in 2020, representing a growth of 12.75%. This growth can be attributed to the implementation of the national innovation strategy and the emphasis on innovation in the new development concept. The Government have emphasized the need to build an innovative country and improve the level of innovation, resulting in significant improvements in the innovation level of the manufacturing industry. Regarding the sustainable development resource utilization level in the manufacturing industry, there is a fluctuating upward trend. This trend is mainly influenced by the disappearance of the demographic dividend and the excessive use of energy, which has led to a resource utilization crisis in the manufacturing industry. However, with the completion of the transformation and upgrading process in the industry, the efficiency of manufacturing industry development and resource utilization has greatly improved. As a result, the resource crisis has been temporarily alleviated.

**Table 7:** Level of sustainable development in manufacturing.

Indicators	2011	2012	2013	2014	2015	2016	2017	2018	2019	2020	average
Composite Index	0.2815	0.2629	0.2688	0.2667	0.2634	0.2697	0.2881	0.3069	0.3190	0.3230	0.2850
Overall Target	0.4192	0.4031	0.4036	0.3997	0.3912	0.3831	0.3851	0.3798	0.3779	0.3668	0.3909
Innovation Level	0.1326	0.0987	0.1259	0.1271	0.1366	0.1593	0.1819	0.2061	0.2295	0.2601	0.1658
Resource Utilization	0.2539	0.2561	0.2373	0.2334	0.2191	0.2265	0.2708	0.3292	0.3503	0.3404	0.2717

**Fig 2:**
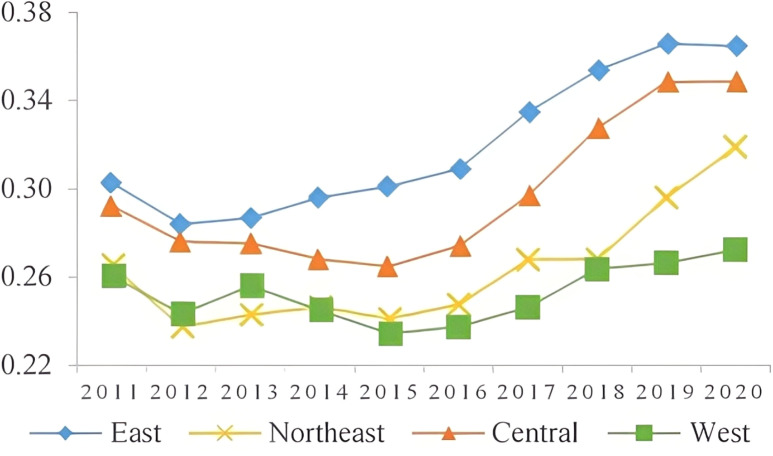
Level of sustainable development.

**Fig 3:**
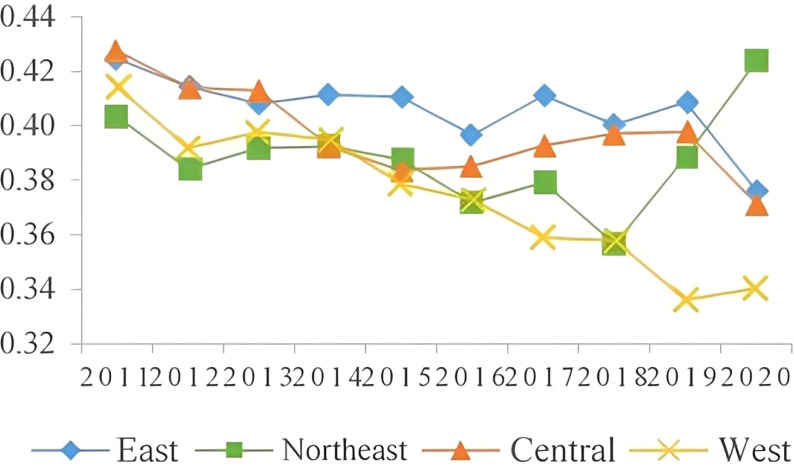
Level of the manufacturing sector.

**Fig 4:**
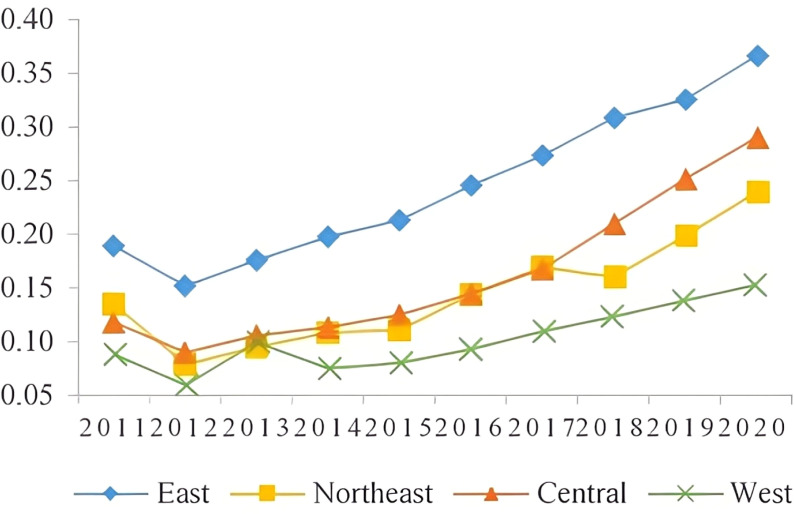
Level of manufacturing innovation.

**Fig 5:**
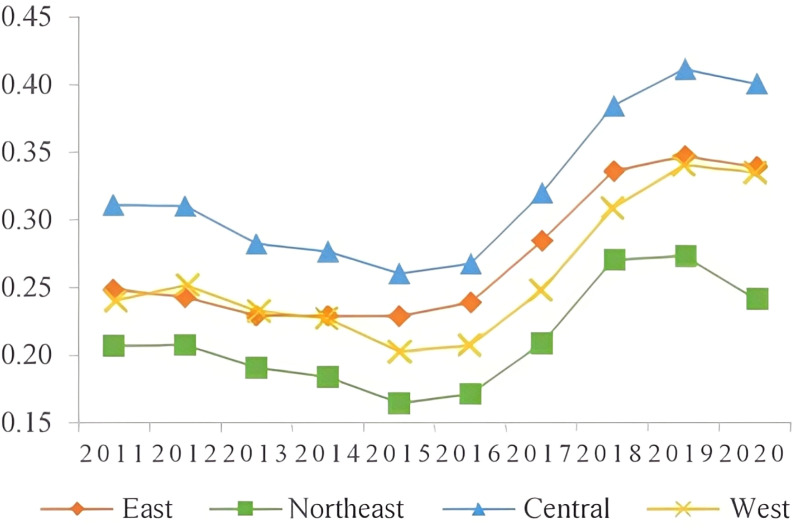
Level of resource utilization.

The results indicate that there is a correlation between the sustainable development level of the manufacturing industry and the regional economic development level. The eastern region, with its favorable geographical location, high-quality labor force, high level of innovation, and efficient resource utilization, has a higher sustainable development level of the manufacturing industry. In contrast, the western region, limited by economic factors and geographic location, has a lower sustainable development level due to a less diverse manufacturing industry and lower modernization level, As shown in Fig 2. The manufacturing industry development target index for all four regions showed a downward trend, with the northeast region experiencing the least decline due to its traditional industrial bases, As shown in Fig 3. The eastern region has a higher level of innovation in the manufacturing industry, particularly in technology-intensive industries, and has shown a rapid upward trend in innovation level, As shown in Fig 4. The resource utilization level of the manufacturing industry has also shown a rising trend, indicating the success of China’s manufacturing industry transformation and upgrade. The government has played a significant role in promoting the shift to intensive resource utilization and improving capital, labor, and energy productivity in the manufacturing industry, as reflected in Fig 5, where the level of resource utilization showed a slight downward trend until 2016, followed by a rapid upward trend.

## 6. Conclusions and implications

### 6.1 Conclusions

This study aims to scientifically define the connotation of balanced, coordinated, and sustainable development of the manufacturing industry. To achieve this, the study constructs an index of balanced, coordinated, and sustainable development of the manufacturing industry and measures the development level of the three industries. Based on this, the study analyzes the temporal and spatial characteristics and evolution law of the balanced, coordinated, and sustainable development of the manufacturing industry. The main conclusions are as follows:

Firstly, the balanced level of most manufacturing industries shows an increasing trend both nationally and regionally. The equipment manufacturing industry, general equipment manufacturing industry, special purpose equipment manufacturing industry, and electrical machinery and equipment manufacturing industry have a higher level of equalization both nationally and regionally. In contrast, the tobacco products industry, petroleum processing, coking and nuclear fuel processing industry, fabricated metal products, and machinery and equipment repairing industry have an overall lower level of equalization. The level of manufacturing equalization in the east is high compared to other regions, while the level of manufacturing equalization in the west and northeast is low overall.

Secondly, the factor structure of technology-intensive industries is highest in the east, relatively high for capital-intensive industries in the central and northeastern parts of the country, and the overall level of development of the factor structure of the manufacturing industry is low in the west. Most of the provinces in China’s manufacturing industry coordination level show a clear upward trend, especially in the east coast and the central manufacturing industry. The coordination level of the manufacturing industry in the west is low, but the progress is most obvious.

Thirdly, China’s manufacturing industry sustainable development level is overall showing a rising trend, with the highest level of sustainable development of the manufacturing industry in the east and the level of sustainable development of the western region relatively low. However, the western manufacturing industry sustainable growth rate is the fastest, and there is a trend of convergence in the level of sustainable development of the manufacturing industry there. The overall goal of the manufacturing industry shows a downward trend due to the decline in economic efficiency of the manufacturing industry. The innovation level of sustainable development of the manufacturing industry has the fastest growth rate, and there is a great improvement in the efficiency of the use of resources in the manufacturing industry under the influence of the improvement of the level of manufacturing industry innovation and the optimization of the industrial structure.

### 6.2 Implications

The findings of this study have important implications for understanding the concept of balanced, coordinated, and sustainable development of the manufacturing industry, and for promoting the high-quality development of the manufacturing industry. Moreover, these findings can also contribute to the national integration strategy and the goal of common prosperity. In light of these findings, this paper proposes the following policy recommendations:

Firstly, optimizing the manufacturing production layout, strengthening the regional cooperation mechanism, and promoting the balanced development of manufacturing. This can be achieved by leveraging the high level of regional manufacturing in the east to lead the development of manufacturing industry in other regions, while also encouraging inter-regional cooperation to optimize the allocation of resources and enhance the radiation-driven capacity of the high-level region. Additionally, it is important to encourage the development of manufacturing in backward regions, coordinate with the western development and northeastern revitalization strategies, and create a favorable industrial development environment to enhance the backward region manufacturing development level.

Secondly, improving the input and output efficiency of the manufacturing industry, optimizing the industrial structure, and enhancing the coordinated development level of the manufacturing industry. This can be achieved by promoting the free flow of factors of production to improve the efficiency of production, and by optimizing the level of coordination of the industrial structure of the manufacturing industry to eliminate barriers to local protection, strengthen inter-regional cooperation and exchange, and build a trans-regional manufacturing industry chain.

Thirdly, improving the level of manufacturing innovation and resource utilization efficiency to achieve social, economic, and ecological goals. This can be achieved by transforming the power mechanism of manufacturing industry development, encouraging innovative behavior, and enhancing the independent innovation ability of manufacturing industry to improve its long-term development. Additionally, it is important to improve the efficiency of resource use through technological progress and optimization and upgrading of the industrial structure to promote the level of sustainable development of the manufacturing industry.

## Supporting information

S1 File Supporting information. (ZIP)
